# Work as an arena for health—Supervisors’ experiences with attending to employees’ sick leave and return-to-work process

**DOI:** 10.1371/journal.pone.0284369

**Published:** 2023-04-14

**Authors:** Nina Elisabeth Klevanger, Lene Aasdahl, Marit By Rise

**Affiliations:** 1 Department of Public Health and Nursing, Faculty of Medicine and Health Sciences, Norwegian University of Science and Technology, Trondheim, Norway; 2 Department of Physical Medicine and Rehabilitation, St. Olavs Hospital, Trondheim University Hospital, Trondheim, Norway; 3 Unicare Helsefort Rehabilitation Centre, Rissa, Norway; 4 Regional Centre for Child and Youth Mental Health and Child Welfare (RKBU Central Norway), Department of Mental Health, Faculty of Medicine and Health Sciences, Norwegian University of Science and Technology, Trondheim, Norway; Caleb University, NIGERIA

## Abstract

**Background:**

Supervisors play a pivotal role in the sick leave process. Although responsibility for sick leave and return to work follow-up is increasingly placed on the workplace in Norway, few studies have explored supervisors’ experiences. This study aims to explore supervisors´ experiences with attending to employees’ sick leave and return to work process.

**Methods:**

This study consists of individual interviews with 11 supervisors from diverse workplaces that was analysed thematically.

**Results:**

The supervisors emphasised the value of presence at the workplace, the need for them to obtain information and uphold dialogue, considering individual and environmental influences on return-to-work and allocating responsibility. Investing time and money was crucial to prevent or reduce the negative impact of sick leave.

**Conclusions:**

The supervisors’ perception of attending to sick leave and return-to-work largely reflect Norwegian legislation. However, they find obtaining information and managing responsibility challenging, suggesting that their responsibilities for return-to-work are perhaps disproportionate to their knowledge on attending this process. Individualised support and guidance on how to develop accommodations based on the employee´s workability should be made available. The reciprocal nature of follow-up described also reveals how the return-to-work process is enmeshed with (inter)personal considerations possibly resulting in unequal treatment.

## Introduction

Return-to-work is a dynamic and complex process in which supervisors play a pivotal role [1, 2]. A recent scoping review found that various stakeholders experience that supervisors’ attitudes and behaviours have a significant impact on sick leave and the return-to-work process [1]. For instance, making employees feel welcomed back [3, 4], creating a positive emotional atmosphere [5] and displaying empathy, acknowledgement [6, 7], support [8] and trust [9] is described as crucial when supervisors relate to employees on sick leave. In addition to being fair [9], honest, respectful and providing validation [10], supervisors also need communication skills [2]. Competencies in areas such as conflict management, upholding confidentiality, knowledge of work tasks and how health conditions influence workability [11] are also identified as necessary. Supervisors are in a position to facilitate accommodations [12–14], let employees participate in decision-making [2], obtain co-worker support and communicate with and coordinate various other stakeholders in the return-to-work process [10]. To manage this, they rely on clear company policies for return-to-work [2] and latitude from senior management [12].

However, employers and employees seem to appreciate different characteristics in supervisors. While employers prefer supervisors who solve problems and allocate responsibility, employees value supervisors who encourage, recognise and protect [7]. Shaw and colleagues´ study on employees´ perspectives on supervisors´ role also suggests that interpersonal aspects are possibly as important as physical accommodation [10]. Several studies have underlined how return-to-work practice is influenced by different perspectives or paradigms of the various stakeholders involved [11, 14–16]. This may result in cross-pressure as supervisors attempt to balance workplace interests and employees’ health concerns [11, 15, 17, 18]. Several other dilemmas have also been identified. For instance, supervisors find it difficult to be unable to meet the responsibilities demanded by policy [17], to secure the safety of employees, and deal with medical practitioners lacking knowledge of work demands [2]. Attending to return-to-work can also be experienced as an emotional burden [2]. Some studies have found that supervisors often feel uncertain although they take on significant responsibility [19, 20] and finding accommodations is recognised as an additional job demand causing strain [21]. Some also struggle with a lack of knowledge and organisational support on how to effectively manage the return-to-work process [22], and find it difficult knowing when to be supportive of employees and when to be confronting and make demands [7]. In addition, supervisors report difficulties in establishing the legitimacy of employees’ complaints [23] and accommodating employees with psychological difficulties is described as particularly challenging [24, 25]. For instance, the invisibility of work impairment, the unpredictable nature of recovery, and difficulties in finding appropriate work tasks are factors that make it challenging for supervisors to support employees with mental health problems [2].

Although many aspects of attending to sick leave and return-to-work seem to coincide regardless of context, the practice within each legislative system necessarily varies [26]. In Norway, employees on sick leave are provided with a full wage for 52 weeks with the employers solely compensating the first 16 days, and the Norwegian Labour and Welfare Administration (NAV) the consecutive period. Therefore, return-to-work legislation has been criticised for leaving both employers and employees with few incentives for assuring rapid return-to-work [27]. However, since 2011 the responsibility for return-to-work has increasingly been placed on the workplace and employees with requirements of return-to-work plans and stakeholder meetings which also include the NAV and occasionally general practitioners. Some studies have explored Norwegian supervisors´ experiences with attending to sick leave and return-to-work [8, 28]. However, the employees in question had musculoskeletal complaints, and studies concerning employees with various diagnoses remain sparse. Therefore, we aimed to explore supervisors´ experiences with attending to employees’ sick leave and return to work process regardless of the employee´s diagnosis.

## Materials and methods

This was a qualitative study based on individual semi-structured interviews with 11 supervisors from a variety of workplaces. The interviews were analysed thematically [29].

### Study setting

The relationship between managers and employees in Norway has been described as generally close and often friendly [30]. Easy and direct communication with rank-and-file characterise interaction, and exercising formal authority (e.g. giving orders) is discouraged [31]. The majority of workplaces are regulated according to the tripartite Memorandum of Understanding on a more Inclusive Working Life (“the IA agreement”). The memorandum states that activity through work is health promoting, and underlines how work withdrawal can be prevented by early implementation of active measures [32].

In Norway, it is usually the general practitioners (GPs) who certifies sick leave. If workability is reduced by less than 100%, graded sick leave, which enables a flexible adjustment of working hours from 20%-100%, is encouraged. Workers receive full wage compensation from the first day of sick leave. From 2011, the NAV introduced a new “Model for earlier follow-up of employees on sick leave” which underline employer and employee dialogue, graded sick leave, workplace- and work task adaptations as well as the active participation of the employee [33]. Within four weeks of sick leave, the employee and employer create a return-to-work follow-up plan, within seven weeks they have a ‘dialogue meeting’, and within 26 weeks the NAV invite them to the compulsory ‘dialogue meeting 2’ where work-related actions are deliberated [34]. The employer can also request that the meeting is conducted earlier. A third dialogue meeting is arranged if needed. If workability remains impaired after one year, it is possible to apply for work assessments allowance or permanent disability benefits; however, these arrangements only account for two-thirds of prior income.

### Research participants and recruitment

This study is based on a convenience sample of participants. The supervisors were recruited in the context of an occupational rehabilitation program which included a workplace meeting [35, 36]. The first author (NEK) contacted the supervisors by phone or e-mail to ask if they agreed to have a researcher present at the workplace meeting and participate in an individual interview afterwards. The aim of the interviews was twofold; to explore their experiences with the workplace meeting (results are published elsewhere [37]) and overall experiences with attending to employees’ sick leave and return-to-work process (the aim in the present study). All supervisors were sent written information about the study and signed a written consent form. From the 17 supervisors asked to participate, ten agreed to be interviewed. In addition, one supervisor was recruited through a rehabilitation therapist (with the consent of their employee) and interviewed without NEK having been present at the workplace meeting to consider if any novel or conflicting experiences or perspectives should be added.

The supervisors were four women and seven men aged between 41 and 64 years old with between 4 months and 30 years’ experience in their current position. Their workplaces included a primary- and high school, department store, laboratory, rental service, security department, hospital department, nursing homes and factories. In this case, the term “supervisor” refers to the role of the employees´ closest leader, such as the manager in a kindergarten, hospital- or nursing ward. Due to the limited number of workplace meetings conducted as part of the rehabilitation program, we have chosen to refrain from matching the supervisors’ characteristics to ensure anonymity.

### Data collection

The interviews were conducted at the supervisors’ workplace between October 2015 and April 2016 by NEK, who has prior experience with performing interviews. The supervisors were asked about their experiences and thoughts on attending to sick leave and return-to-work generally. The questions included what they found to be important elements in the process and what hindered it, and what dilemmas they had experienced when attending to sick leave and return-to-work. They were also asked to reflect on themes such as cooperation, openness regarding the reason for sick leave and the relation between work- and private life. The interviews lasted approximately one hour and were audio-recorded and transcribed verbatim.

### Data analysis

The interviews were analysed thematically [29] within a symbolic interactionist understanding [38]. As such, the individual experiences and statements of the supervisors are understood as intimately linked to the interpretational process arising from interaction with others [38], in this case, their employees. Two researchers with social science/public health backgrounds and one with medical- and public health research background contributed to the analysis. First, NEK and MBR read all interviews, noted first impressions and discussed them. NEK then coded all, whereas MBR coded two interviews to compare and discuss similarities and discrepancies, resulting in a thematic map including all codes (Table 1). This map was then discussed with LA, who also read two interviews, and two themes were subsequently combined and one retitled. During the analytic process, preliminary results were discussed in two different research groups where the authors are members. We included quotations with pseudonyms to exemplify or nuance the analytic text of the results, and some minor adjustments insignificant to meaning were made to improve readability.

**Table 1:** Thematic map.

**Encouraging work presence**	• difficulties in returning increase as sick leave proceeds • work presence promotes health, ownership and a sense of belonging • underlining the value of presence is important, either by being reassuring (“take your time”) or strict (“what can you do”)
**Obtaining information and upholding dialogue**	• early-stage contact and continuous dialogue are important to show employees they are appreciated • information about causes of sick leave is necessary to find accommodations and determine if an absence is work-related • which information to request and how is difficult, cautious approaches are the safest
**Considering individual and environmental influences on sick leave**	Sick leave must be approached: • individually—factors such as employees’ personality, openness, and attitudes toward sick leave must be considered • in the work environment—promoting well-being/thriving and appropriate attitudes toward sick leave is important, delimiting if sick leave is an individual or systemic problem is difficult
**Allocating responsibility**	• supervisors´ primary responsibility is finding appropriate work accommodations • responsibilities are unevenly distributed: • employees should be held more accountable for making contact, finding accommodations, and their health situation • GPs are gatekeepers but possible allies if dialogue improves • occupational health services can address private difficulties
**Investing to reduce the negative impact of sick leave**	• attending to and preventing sick leave is time-consuming and expensive • preventive measures include monitoring to detect problems, formal and informal conversations, surpassing budget, granting paid leave

### Ethical considerations

The Regional Committee for Medical and Health Research Ethics in Central Norway approved the study (no.: 2014/2279). All participants signed an informed consent form before the interviews commenced.

## Results

*Eve: When someone is about to become sick listed, it is so beneficial to first approach the supervisor. Then we can explore: “What can you possibly….”. You should get to bring your story forth, and what you want to keep private, you keep private. But if you can say something about your sickness situation, your problems… At least how the workplace may help you? And then I find work to be a matter of health. In your sickness situation, work can help you to get back.*

*Interviewer: Do many perceive work in that way once they are on sick leave?*

*Eve: No. I think way too many let their key to work go. And become very private. So I believe I… That you as an employer need to do a large piece of work to keep in touch and sign them on.*

                        (Eve, primary school)

### Encouraging work presence

The supervisors spoke of work presence as the most crucial measure to incite full return-to-work and a common statement was that difficulties in returning increase as sick leave proceeds. Being absent was said to lead to feeling useless, and some supervisors described that they even believed staying at home could worsen health difficulties as the employee then spent more time noticing, e.g. sensations of pain and discomfort. Work, on the other hand, was explicitly framed as health-promoting, and many supervisors spoke of giving employees the *opportunity* to be at work while on sick leave or *letting* them work. For instance, for one supervisor work represented a source of energy to deal with private issues causing sick leave:

*I believe that when we experience difficulties of any kind in our private life–if you find work interesting enough to gather nourishment and energy, can be psychologically and physically present when you are at work, and manage to put things aside–then you gather energy to better manage difficulties. I believe that if you just go: “Pass” and stay at home with those difficulties, then you just dig yourself deeper. But if you can go out and gather some energy (…) then you have built yourself up with lots of vitamins to move on with those difficulties.*

                        (Eve, primary school)

In addition to being health-promoting, the supervisors described being present at work in some way or another, as instrumental during sick leave. Some described working as rewarding in itself due to the primacy of work in society and keeping in contact with the workplace was perceived to promote ownership and a sense of belonging. The supervisors saw maintaining social attachment to work as essential to avoid the difficulties of returning if one had not been in contact with colleagues and supervisor for an extended period.

The supervisors told of different approaches to incite work presence. Some spoke of telling employees they were wanted back in due time while simultaneously underlining that being present was predominantly in their best interest. Other supervisors described a stricter approach, demanding presence at meetings or workplace visits to keep updated on frequently changing procedures:

*It is important to maintain the social- and the professional aspects. If you are unable to come in and read emails and keep updated, you must be very sick. And because changes are made weekly, it is very important to keep updated at all times in this line of work (…) You can do that (keep updated) if you are on sick leave, I believe. You just go to work, sit down and read emails, converse a bit, observe. You don’t need to do more than that to keep updated.*

                        (Trond, security department)

Some supervisors underlined that the value of being present was somewhat dependent on the cause of sick leave and that employees sometimes should be reassured that they should take their time getting well. However, most supervisors seemed to believe that employees were able to perform some activities at work, regardless of diagnosis and being full time sick-listed. Several believed GPs provided 100% sick notes due to a lack of knowledge of their patients work situation. Therefore, some supervisors told that they could request meetings with the employees to explore whether they believed they were able to do some tasks at the workplace after all, and which accommodations they needed to do so. Although graded sick leave was described as ideal, several supervisors felt frustrated by how employees and GPs interpreted the employee’s ability to perform within a given percentage:

*If I get in a sick leave, I sometimes call the GP to figure out: “What is this? What do you think? Are we talking about the same thing? Do we have the same understanding?” (…) It has to do with making the GP aware as well, especially what they have said regarding: «Yes, you can work 50%». That means you can be present 100% at work but deliver 50%. Not many employees think like that when they get a sick note of 50%.*

                        (Liv, nursing home)

### Obtaining information and upholding dialogue

All supervisors underlined the importance of obtaining relevant information and continuous communication with employees on sick leave to help them return-to-work. Contacting the employee and asking how they were doing was described as ways of showing employees that they were cared for and wanted back.

*The most important thing in my mind is to have a dialogue, a continuous dialogue. If the employee delivers a sick note and I don’t contact them for two months, it is doomed to fail. It has to do with showing the employee that the employer actually cares for the employee. (….) Is the employee unable to work at all? We have those cases too. That’s how it is. Then the workplace is still to have a dialogue and contact with the employee. Inviting them to department meetings, inviting them to: “Hey, we are having Friday coffee, we have bought pizza, can you come by?”. Calling to see how they are doing: “How are you?” I believe that it is alpha and omega.*

                        (Anders, factory)

The supervisors argued that information about the employee’s health difficulties as well as the causes for them was first and foremost an important tool in finding appropriate work accommodations. For instance, knowledge of cause for sick leave was described as crucial to secure that accommodations were based on the true requirements of the employee to return fully to work, and not convenience. One supervisor described that most employees were open and that the more they shared, the easier it became to instil accommodations. Information was also described as something that could improve the relationship with the employee and enable colleagues to provide needed support.

However, the decisive type of information mentioned by most supervisors was whether the cause of sick leave was work-related or not. This seemed to determine if the supervisor felt that s/he had any influence or responsibility for making changes at the workplace. If not work-related, most supervisors still valued information as they believed they could develop more appropriate accommodations. Although a few pointed out the futility of demarcating between work-related and other causes for sick leave as they nevertheless were intertwined, most supervisors described remaining rather passive if factors outside their reach caused sick leave:

*«Because of my home situation, it is hard going to work right now», or “because this and that”. Then that is that. And then I don’t do anything. It is like that then; we rather have a meeting later instead.*

                        (Anna, hospital department)

While all supervisors described having knowledge of the employees’ health difficulties as essential, they simultaneously expressed insecurity in which information to request and how. They were well aware that they could not probe into employees’ diagnoses and were reliant on their willingness to share information. All told of difficulties when approaching employees at some point in time. One supervisor described looking forward to attending an upcoming course on how to encounter employees on sick leave, finding his limited vocabulary to negatively impact interactions:

*Joakim: I don’t have a lot of education; my vocabulary is perhaps too limited, so things can be misunderstood. That can be a bit scary.*

*Interviewer: Yes, because you talk past each other, or…*

*Joakim*: *Yes*, *because if the reason for your sick leave is something psychological*, *you are a bit* wary *as we say*, *and then you might interpret some things*. *And then it is easy to interpret them a bit wrong*, *you know that from your own experience*. *Therefore*, *it can be interesting to have a meeting with the NAV*, *or attending a course*, *to know a bit more about which questions I can ask*, *which not to ask and how to ask those questions*.

*Interviewer: Yes, because that is not easy.*

*Joakim: No, my goodness, it is not! You can put your foot in it (…).*

                        (Joakim, department store)

If an employee refrained from providing any information, most supervisors assumed that causes were “private” or “psychological”. Although several underlined employees’ right not to share diagnosis or cause of sick leave, all supervisors seemed to expect some information. If employees refrained from providing any, one described becoming suspicious while another believed the employee then had a greater responsibility for providing information on possible work accommodations. Most supervisors told of experiences with employees holding back information, and when so, they described different approaches. Many referred to “fishing” for the cause of sick leave by enquiring how they could contribute without asking directly for causes. Another approach was to accept the lack of information and instead make time for conversations with no other specific goal than being a fellow human being and keeping the workplace “fresh in mind”.

However, most supervisors described feeling frustrated when lacking information, and several spoke of the value in being explicit when addressing the employee. Speaking one’s mind or stating genuine opinions was portrayed as beneficial since it made it easier to make demands and find appropriate solutions faster. One supervisor said that as long as trust characterised the relationship with the employee, it was best to be direct. Some also described having a way of reading their employees and knowing how to proceed due to their long experience as supervisors. Still, these supervisors told of experiencing difficulties when approaching employees in the past and that manoeuvring the boundaries of the individual employee and legislative duties could be troublesome. As such, they underlined the need to be cautious due to never being entirely sure of the background for the employee’s struggles, and since being direct possibly had the reverse effect of intended as the employee could respond negatively.

### Considering individual and environmental influences on sick leave

Many supervisors underlined how both individual differences, such as tolerance level and personality, and the workplace environment influenced sick leave and return-to-work. For this reason, they described that it was necessary to have an individual approach while also ensuring a work environment fostering appropriate attitudes.

Many supervisors referred to how attitudes acquired throughout life influence when employees define themselves sick, how they relate to being on sick leave and also work ethics in general, and as such, viewing work absence as a question of morality. Although described as exceptions, several told of experiences with employees they suspected were not genuinely sick. One supervisor also reflected on the possibility of sick leave being culturally defined, questioning if high absence in Norway compared to, e.g. China and the US is a result of allowing oneself to be sick, while another believed she observed generational differences. As such, the supervisors occasionally framed sick leave as relative instead of an objective state of inability to work. This view also became apparent regarding tolerance level, which was described as both individual and dependent on the employee’s current health situation. The supervisors appeared to predominantly perceive sick leave as an individual issue in need of a personal approach. They underlined the futility in using a blueprint for return-to-work on everybody, and the employee’s personality and level of openness were commonly referred to as important factors when deciding how to proceed. A few also described how their impression of the employee’s attitude towards sick leave and return-to-work affected how they encountered them. In one interview, the supervisor told of rewarding an employee on sick leave due to what she considered “real” musculoskeletal problems with flowers because of her prior efforts and explicit wish to resume work.

Several supervisors also underlined how workplace culture influences sick leave attitudes, therefore working actively to create an environment that prevented sick leave and fostered presence once on sick leave. The supervisors identified employers and colleagues as key agents apart from themselves in this regard. Wellbeing/thriving (Norwegian: “trivsel”) at work was identified as essential in prohibiting sick leave and promoting return-to-work:

*We try to create the best possible workplace regardless of being an IA workplace or not, it doesn’t matter. We want people to thrive at work. Because they want to be present if they thrive. That is the first commandment. And the work environment, the psychosocial work environment, that is important. Thriving with the rest of the staff, but also experience that they are doing something useful.*

                        (Ingrid, laboratory)

Work task variations, room for making mistakes, involvement in decision making, independence and freedom were aspects believed to foster wellbeing at work. Also, some supervisors described that they worked actively to develop a work culture promoting appropriate attitudes towards sick leave. This entailed to spur reflections on sick leave and interaction with colleagues, and making it evident that employees should come forth with health concerns as soon as they arose:

[We] *have attention constantly among the staff on*: *“How should we approach someone who is at work with accommodations*?*”*. *A good example on what to not say*, *which is probably coming from a good place but might have the reverse effect*, *is colleagues saying*: *“Oh*, *I can see that you are in such pain*! *Shouldn’t you be at home*? *Shouldn’t you be on sick leave*?*»*. *Instead*, *the focus should be on*: *“I see that you are struggling a bit and are in a bit of pain*, *but it is so good that you are able to be here*! *We are so happy that you are able to be present at work*!*”*.

                        (Peter, nursing home)

The individual and environmental influences on sick leave also presented the supervisors with a dilemma. Several pointed out that it was sometimes difficult to know if sick leave resulted from work strain that was objectively too high, or from the employee having a low tolerance level. In turn, this had consequences for how the supervisors believed they should accommodate. For instance, one supervisor questioned to what extent she should approach sick leaves resulting from e.g. work-related stress as an individual or a systemic problem:

*We have created a work-life which is quite stressful to very many employees. (…) If ten out of twenty reports that demands are too high or it is too stressful, you cannot merely say: “pssh, go home and learn stress management, it will be ok” (…) Should we address them or the system or both? And then it is relevant to find out what is suitable for you and not, and in some cases conclude: “Okay, this is not the workplace for me, working here makes me sick, and then I need to find something else”. I believe we have a personal responsibility to not just say: “Well, I cannot manage, someone else has to find out what I should do”.*

                        (Liv, nursing home)

### Allocating responsibility

The supervisors frequently referred to the legislative agreements defining their role and generally described taking this obligation very seriously. Finding appropriate work accommodations was commonly referred to as their primary responsibility when attending to employees on sick leave. However, most supervisors also experienced that responsibilities in the sick leave period were unevenly distributed, with a general lack of attention to those of the employee. For instance, many told of frequently having to take on the responsibility for initiating and maintaining contact, although legislation describes this as a joint responsibility.

Several supervisors believed employees should be held more accountable for e.g. the sick note, finding suitable accommodations and their health situation. Some seemed irritated with employees merely referring to their sick leave as their GPs assessment:

*(Sigh) It is a bit like that when they return: “My GP said…..”. And then I often respond: “Wasn´t it you who told the GP?”. That is more honest, and then I find it very interesting: “What did you tell the GP?”. Because we put our lives in the hands of the GP and he listens to us too, and then it has to do with taking responsibility for what you brought to the table. What did you share? And what was the result?*

                        (Eve, primary school)

The supervisors also spoke of GPs as the gatekeepers to (graded) sick leave due to their role as sick leave certifiers. While some called them too kind, others believed they simply lacked the knowledge to assess which work tasks an employee could perform. Many spoke of wanting a closer dialogue, and some told that they sometimes called GPs to discuss an employee’s sick leave if they believed it was incorrect. One supervisor also provided GPs with follow-up plans developed in collaboration with the employee in an attempt to accommodate the GP. As a result, GPs could become possible allies since they, when presented with multiple tangible suggestions, often found some appropriate. Bypassing the employee and reaching out to the sick leave certifier was thus described as a possible strategy if employees refrained from taking their responsibility for transmitting information on possible accommodations.

*When they have decided to be absent, they are absent. That is how it is. It is them and their GP. But if I suspect this, I can request an early dialogue meeting with the GP, I sometimes do. Sitting there with the GP and ask: «Is there any workability? We can accommodate, it is possible to be in the kitchen for the day, washing cups or making food, sitting down talking to patients or…”. […] If the issue is a broken toe, it is difficult for a GP to decline that they can sit and read the paper with a patient or such things. Here, you can be of use for many things.*

                        (Anna, hospital department)

Some supervisors also underlined how employees could often be held more accountable for the results from not using available technical aids, one describing that employees sometimes developed work-related stress due to lack of time with the residents at the nursing home. Nevertheless, she also believed that the employee should learn how to cope by demarcating for what s/he could be held responsible him/herself:

*Even if we doubled the staff, I believe you would still have the feeling that you could have done more. It has to do with being a bit hard on yourself and learn to say: “I did a good job today. I did what I could, and others set the premises for how much I can do”. If you know that you have structured your day and did all right… Then again, the challenge is that we are different in that respect. A lot of us would benefit from attempting to let work go once returning home.*

                        (Liv, nursing home)

The supervisors described work and private life as intimately linked, and although most stated that their responsibility stopped at the doorstep of the workplace, some explained that they saw the need to help employees find solutions to private difficulties. Here, some contacted the occupational health services at their workplace. These services were portrayed as having a holistic approach, being more competent in handling private issues and having the status of a neutral third party in the return-to-work process. Although several supervisors believed the occupational health services were capable of thematising private issues they themselves were not, a few nevertheless did. Some described that they then asked employees about their health situation, and in some instances encouraged them to reflect upon whether they should reduce activities in their spare time to prioritise work.

Some supervisors also underlined that it was the employee’s duty to approach them before sick leave commenced and ask for accommodations. One described that he believed this could reduce the severity and length of sick leave:

*If they turn up with a sick note without having discussed it with me, if they have struggled for some time, I believe they have not upheld their duties to inform their supervisor of their difficulties. […] When that happens, I talk to them: «Why haven’t you mentioned this to me before? What have we agreed on in our department? How should we handle these things?». If you get started earlier, you could possibly avoid a total collapse, you address it at an earlier stage and get better.*

                        (Peter, nursing home)

### Investing to reduce the negative impact of sick leave

The supervisors described both attending to and preventing sick leave as essential, however, time-consuming and expensive activities. Time and money were framed simultaneously as both scarce resources and well-spent investments to reduce the length or impact of sick leave or avoid it altogether.

Many supervisors underlined the need for being attentive to signs indicating that employees experienced difficulties possibly leading to sick leave and, if necessary, take the time needed to attend to these difficulties. In doing so, the supervisors told of various approaches. One described monitoring employees’ production since difficulties in keeping up with the phase could be a sign of other problems:

*We have started counting how much they can get done, and we measure all employees. Measuring is a bit scary though, keeping an eye out and counting can be misused or be interpreted as misuse. So we have turned it around, employees tell how much they believe they can do and if they are able to reach their daily goal instead. And then we soon find out if they have periods where they are far below what they usually manage.*

                        (Ingrid, laboratory)

Another requested employees to call her directly if sick, describing this as a dual approach serving as both a preventive measure and a means to keep updated and meet employees appropriately afterwards. However, the supervisors usually described more personal approaches, taking time for face to face conversations with the employees. These conversations could be structured and instigated by the supervisor, such as the “health conversations” a supervisor in a primary school described having with employees on recurrent sick leave:

*Health conversations? I have those with frequently absent employees. If I pick up on employees having troubles in their life I ask: “Can we have a health conversation?” And to me, that concept is expanded, it is something else than an appraisal, and more than just: “Can you pop into my office?”. I tell them what it is about and make my intention clear: “It is not to be nosy; it is to genuinely find out how I may support you when seeing that you have it difficult. Don´t tell me more than you want to, but if [some information] could help me to understand you, to accommodate you better at work so that we don´t put unnecessary pressure on you, [then I can] be your ambassador towards colleagues to gain more understanding from them. That is why we are having this conversation”.*

                        (Eve, primary school)

Conversations could also be spontaneous, and several supervisors told of having to let everything else go and prioritise an employee wanting to discuss issues. Following up employees already on sick leave with meetings and keeping in contact was often labelled as time demanding. One supervisor described this as a dilemma, possibly leading to differences in how employees were treated in the return-to-work process:

*I believe it is very dependent on how much the employer instigates activities and meetings and treatment. Although it is demanding, it is possible. I find that it takes time, and in the cases in which we can simply bring in a temp, and then everything is solved, it is easy to do so. A truck driver, for instance, we can find them in a minute. And the work proceeds, it gets done. But the NAV is quite agile when it comes to sick leave, so we work actively on that. But it is a bit dependent on the caseworkers, how much you push to find solutions to get activity.*

                        (Einar, factory)

The supervisors also highlighted how economy restricted their opportunities to attend optimally to sick leave and return-to-work. Whereas one supervisor stated that only creativity sets the limits for accommodations, most supervisors described that their budgets related to sick leave were limited or “not realistic”. When referring to economic restraints, one described how cutbacks led to additional work tasks for employees who in turn felt stressed and overworked. Also, several supervisors explained that not having the means to replace those on sick leave meant that the rest of the staff, in turn, were at risk for developing health difficulties, creating a vicious circle of sick leave and stressed co-workers.

Although the supervisors largely agreed that they had scarce means, most spoke of economy in terms suggesting that they had accepted their limited budgets and concentrated for instance on applying for compensation from the NAV, redistributing staff and having apprentices filling in. One also told of the need for assuming a larger timeframe for return-to-work, and that she accommodated all employees on sick leave although this resulted in a deficit, believing that it was profitable in the longer run. Another approach was to grant the employees “welfare leave”; one or two days off with full pay if in need for rest. The supervisor underlined that simply offering such an arrangement could make a difference:

*The employees have many challenges, some you know better than others too, and you know their limits. I have one employee who is struggling a bit, have been for a while. I have regular conversations and tell her: “Come see me if you feel it is too much. Have a long weekend, take Friday off, then you have three days to recover. Start afresh next week”. That is to prevent sick leaves. Just knowing that “I see you; I understand that you struggle, and you can approach me to let me know”.*

                        (Anna, hospital department)

Several supervisors also mentioned that they sometimes felt conflicted between responsibility for the employee on sick leave and the other employees. However, neither time nor money were described as zero-sum games; instead, they were portrayed as investments believed to provide a surplus at a later point. As such, return-to-work was portrayed as a complex and time-consuming process requiring attention and resources at all times; to prevent the onset of sick leave, facilitate presence while on sick leave and avert recurring sick leave in the workplace as a whole.

## Discussion

This study aimed to examine supervisors’ experiences with attending to employees’ sick leave and return-to-work process. The supervisors emphasised the value of presence at the workplace, their need to obtain information and uphold dialogue, as well as considering individual and environmental influences on return-to-work and allocating responsibility. They also underlined how investing time and money was crucial to prevent or reduce the negative impact of sick leave.

### The emphasis on presence

First and foremost, the supervisors’ perceptions of presence, graded sick leave, early contact and activity during sick leave and prevention coincide with the underlying premise of the IA agreement legislating most Norwegian workplaces [27]. As such, the result of the current study might suggest that the IA agreement is well implemented. Although most supervisors noted that some health difficulties warrant full sick leave, the overall call for presence was striking. Although the benefits of early work resumption have been demonstrated, workplaces are not necessarily able to offer accommodations that safeguard employees´ health [39], and studies in other legislative contexts have also found that graded sick leave may be challenging to employees who must manage to be present while sick [40, 41]. Since employers’ actions in return-to-work can be understood as ‘rituals of legitimation’ [18], the expectation of employees being able to perform work tasks despite being on sick leave may serve as a way of delegitimising their sick leave. Therefore, the strong emphasis on presence and workability could be problematic and should be approached according to the employee’s individual needs.

### The challenge of obtaining information

Obtaining what they considered relevant information to facilitate return-to-work was occasionally challenging to all supervisors, which is also found in a prior Norwegian study [28]. Instances in which employees did not provide any information about the cause of their absence were identified as particularly demanding, and their difficulties were then often interpreted as “private” or “psychological” in origin. Studies from other contexts have also described how supervisors often find it more challenging to support employees recovering from mental health problems due to their perceived need for more empathy, longer recovery time, unpredictability and being difficult to manage [2]. Since supervisors often lack knowledge of symptoms connected to mental health problems, and it is unlikely such symptoms improve once an employee has returned to work, it has also been pointed out that a clear definition of the employee’s functional limitations is necessary [42]. The supervisors in the present study described “fishing for information” or becoming passive in cases where they lacked information, and to request information from the employees’ GP if they suspected the sick leave was undue, approaches that could be unproductive. It seems beneficial to increase supervisors’ knowledge about common mental health disorders, symptoms and possible consequences for work and daily function. This could help improve their skills in communicating with these employees, as well as equip them in finding suitable accommodations and adjusting expectations according to their specific context.

### A shift towards workability

The results reveal that the supervisors seemed to expect and appreciate information concerning the causes of absence to develop appropriate accommodations. However, in Norway, sick leave follow-up represented by the NAV and IA agreement is primarily concerned with determining employees’ workability. A focus on workability can bypass the problem of employees having to share confidential health information that supervisors must handle. Although it is essential to maintain the right to refrain from disclosing the diagnosis certifying sick leave to employers, the flow of information between different stakeholders regarding workability should be facilitated. This is especially important since GPs work capacity assessments are highly complex, to a large extent tacit [43], and since stakeholders´ divergent assessments of work capacity and sickness certification may lead to contradictory messages that hamper recovery [44] or resistance towards an early return [45]. In this study, GPs were identified as having a pivotal role as gatekeepers for sick leave, while simultaneously lacking the appropriate knowledge of their patient’s workplace, which mirror NAV caseworkers perspective of GPs [46]. Norwegian supervisors have previously pointed out the need for closer cooperation with GPs to facilitate return-to-work [8]. Providing the employee with a premade list of possible work accommodations for their position to discuss with their GP, as some supervisors in the present study did, is one solution to assist GPs in their assessment of patients’ workability. Additional courses and information on providing relevant and operational health information to supervisors regarding their patients’ workability at their specific workplace is another. Simultaneously, we suggest that guidance on finding accommodations based on workability is made more readily available for supervisors to diminish their preoccupation with diagnoses and facilitate communication with employees who choose to refrain from sharing information. An important role of future research is to explore how various return-to-work actors can address workability without trespassing the boundaries of requesting information on the causes for sick leave.

### Individual and cultural approaches

The results revealed that the supervisors recognised how both personal and cultural influences on sick leave necessitated a dual approach to sick leave and return-to-work. However, they mainly spoke of preventing sick leave through attending to the workplace *culture*, describing few tangible changes to e.g. work organisation or *structure*. Even the supervisor highlighting the dilemma of knowing if causes for sick leave resided in the individual worker or the workplace structure ultimately spoke of holding employees accountable for their health, and that this served as a solution for reducing sick leave. As such, sick leave was predominantly framed as an individual problem in need of an individual approach. Previous studies have also found that supervisors tend to attribute reasons for sick leave primarily to functional limitations, individual circumstances or to the personality of workers, even when working conditions are recognised as demanding [18, 47], and workers are diagnosed with work-related stress [11]. Refraining from implementing workplace interventions to reduce stress has been connected to managers’ attempt not to be perceived as failing or weak [47] or avoid guilt [11]. Also, addressing psychosocial factors at work such as relationships to colleagues and supervisors and work demands requires more complex involvement of employers than e.g. gradual return and reduction of workload [42]. However, these factors are perhaps especially important to address since they are linked to both the onset and development of psychological symptoms, will still impact employees upon return, and are of benefit for the workplace as a whole.

### The burden of responsibility

The supervisors’ emphasis on addressing the employees’ responsibility for safeguarding their own health can also be interpreted as an attempt to redistribute some of the responsibility placed on supervisors. Although believing return-to-work to be a joint responsibility, the supervisors told of spending much time on sick leave follow-up—taking on employees´ duties and feeling insecure about how to proceed if they lacked information. Employers/supervisors are recognised as having the central place in the return-to-work process alongside the employee [48]; however, several studies have identified that they struggle with their responsibilities for return-to-work [15, 17–20]. For instance, employers have been found to perceive accommodation management as a considerable addition to their other duties, feeling ill-prepared, even when given guidance [21]. Norwegian supervisors have also been identified as insecure in how much they should accommodate, and often end up stretching too far in an effort to stay well within legislative boundaries [46]. Overall, this suggests that supervisors’ knowledge of attending to the complexities of the return-to-work process is insufficient relative to the high degree of responsibility they are provided.

The IA agreement, regulating the majority of Norwegian workplaces, has been recognised to place the problem of sick leave on the workplaces, emphasising accountability, prevention, dialogue and accommodations, thus paving the way for a socially anchored concept of sickness and health [27]. Within this discourse, function is not solely dependent on medical issues; instead, factors such as relations and workplace accommodations are given precedence in understanding what affects sick leave. Whereas recent reforms in the Nordic countries have been described as less likely to promote a strong workplace approach [48], responsibility for return-to-work in Norway has increasingly been placed on the supervisors and workplaces of workers [49]. This shift towards “self-reliance” in which subjects (i.e. workplaces) are given the responsibility of regulating or governing themselves has elsewhere led employers to assume disciplinary roles previously filled by government or compensation board personnel [40]. Imposing the responsibility for return-to-work to the ‘ecology’ of each local workplace may undermine the policy’s original intent [40], and it has been suggested that increasing employers’ role in return-to-work follow-up should not occur without simultaneously providing employees support [45]. The results of the present study indicate that many supervisors could gain from additional competence and support as the responsibility of attending to sick leave and return-to-work occasionally falls heavy on many of them. In Norway, the NAV provides support in sick leave follow-up work. However, apart from assessing the supervisor and employee´s activities eight weeks into the sick leave, the NAV is not an active part until the 2^nd^ dialogue meeting at 26 weeks. The employer can apply for expert assistance from NAV in case of long term and/or frequently recurring sick leave; however, the employee must agree that such assistance is appropriate. In our material, only one supervisor mentioned using the NAV as a resource in sick leave follow-up, and the supervisors´ difficulties predominantly seemed to emerge at the beginning of the sick leave trajectory. Since supervisors are expected to manage follow-up by themselves, the bar is possibly set too high for supervisors to request such assistance, and it is important to question whether the expectations of supervisors are reasonable and how their present role affects the return-to-work process. It is also interesting to note that the supervisors did not address any lack of support from their own employers, and as such, they seem to assume the responsibility. Based on the results of this study, we propose that competence enhancements should be accompanied by support that is both low-threshold and readily available. Since NAV caseworkers experience that they lack the capacity to be an active part at an appropriate stage in the sick leave trajectory [46], providing such support seem to require additional resources.

### The personal and reciprocal character of return-to-work

The results of the present study also suggest that the return-to-work process is highly reliant on (inter)personal factors and moral considerations. Although the supervisors emphasised the cause of health difficulties, they also maintained that they approached employees according to the personality of the employee. Follow-up during sickness absence was also described as reciprocal in nature, revealing how the process is enmeshed with prior role fulfilment, the status of the employee as well as the employee-supervisor relationship. Several studies have underlined the need for interpersonal skills in the return-to-work process [2] and Eakin et al. (2003) use the concept of ‘moral economy’ to describe how social interaction can be flavoured by moral instead of formal, contractual relations. As such, “moral expectations and commitments between workplace parties are exchanged or traded, and moral ‘capital’ can be ‘banked’ or stored up as a resource” [40], p. 24. Especially in small businesses, the relation between workers and employers can be characterised by a blurring of the ‘we-they’ identities, a relatively small social status gap, as well as strong norms of independence, reciprocity and mutual obligation [40]. In the present study, several supervisors described a reciprocal view of return-to-work in connection with highly skilled employees with long-term employment, or employees showing a high degree of work ethics. Other studies have highlighted how worth is connected to productivity in paid work [50], and maintaining a strong work ethic is important in contemporary welfare states due to the expenses represented by work absence—probably even more so in countries where legislation protects sick workers’ income for as long as in Norway. Although the supervisors told of malingering workers as exceptions, most nevertheless seemed to consider them when describing their own dilemmas on facilitating presence. The supervisors’ considerations suggest that they differentiate between low and high-value employees, an action also identified by NAV caseworkers who observed supervisors´ engagement to be far lower for easily replaceable employees than for those with valuable competence [46]. One interesting aspect is how one supervisor describes how the NAV does not differentiate between employees in the same way, thus securing unbiased follow-up of all employees. Placing the responsibility for return-to-work follow-up on the workplace entails that the process to a greater extent is dependent on the supervisor-employee relationship. As such, the NAV could be an important actor in securing employees a just treatment relieved of personal or professional favouritism, given their inclusion in the return-to-work process at an early stage.

The results also show that although the supervisors agree that their budgets for attending to sick leave are limited, they seem to have accepted this, rather concentrating on making arrangements within the workplace or applying for compensation from the NAV. Here, the Norwegian context seems crucial. Norwegian employers only compensate the first 16 days of absence, and what has been labelled generous welfare arrangements seems to compensate for the lack of workplace sick leave budgets, leaving supervisors content. The sick leave legislation has been criticised for presenting employers with poor incentives to help employees return-to-work [51]. However, the supervisors in the present study seem to value and attend to their employees on sick leave, applying several strategies for keeping them present at work.

Strengths and limitations

This study comprises interviews with supervisors from a variety of occupations with different length of experiences as leaders. Despite these differences, the supervisors shared many experiences of what they found important and challenging when attending to sick leave, suggesting that the results are, to some extent, transferable. The legislative- and cultural setting of the study necessarily limits the relevance in other settings. For instance, the role of GPs, the compensation system governed by NAV and the strong work culture entail that some aspects of the study are not readily transferable to other contexts. However, aspects such as difficulties concerning graded sick leave, psychological causes for sick leave and the interpersonal aspect of return-to-work are found elsewhere, suggesting that the supervisors´ experiences in the present study also reflect other legislative contexts. The material is based on a convenience sample in which participants were recruited in the context of another study. The supervisors had already agreed to partake in a workplace meeting as a part of an occupational rehabilitation program where one of their employees participated. As such, they were perhaps especially interested and knowledgeable in the topic and practice of attending to sickness absence. This poses questions about both the representativity of the sample and the transferability of the results. Furthermore, the results are a reflection of the supervisors´ own relationship to work. Due to their position in the workplace, supervisors might attribute personal meaning to their work and have distinct work identities which not all employees can be expected to share. The researcher who conducted non-participant observation of the workplace meeting also conducted the interviews (NEK), and in the interviews, the supervisors were asked to share experiences with these meetings as well as general reflections on how to attend to sickness absence. This affected the results and analysis in different ways. For instance, the supervisors’ statements sometimes referred directly to the employee who also participated in the workplace meeting, sometimes describing how his/her case resembled or was different from others. In the analysis, we therefore considered if the supervisors´ experiences or actions seemed like exceptions or commonplace instances for how they attended to employees on sick leave. Agreeing to participate in an interview after the workplace meeting could also be a result of wanting to justify/amend their appearance in these meetings. Another limitation is the duration since conducting the interviews. However, Norwegian sick leave legislation has not changed significantly within the timeframe from the interviews, so it is reasonable to assume that the supervisors´ experiences are relevant.

The different backgrounds of the authors and the input from two inter-professional research groups strengthen the interpretations and affirm the results of this article. Whereas this material provides insight into the supervisors’ experiences with attending to sick leave, it is solely based on the supervisors´ statements. As such, their actual responses to sick leave remain unknown to some degree. Conducting participant observation at the workplaces or interviewing pairs of employees and supervisors to explore any commonalities or discrepancies in experiences would provide valuable information into how supervisors attend to employees’ sick leave process. Future research should include such methodological variety to enlighten the field.

## Conclusions

This qualitative study explored supervisors’ experiences with attending to employees’ sick leave and return-to-work. The supervisors’ experiences with the value of presence, graded sick leave, and early contact largely reflects Norwegian sick leave legislation. However, all supervisors reported difficulties in obtaining information and having the primary responsibility for return-to-work follow-up, suggesting that their responsibilities are perhaps disproportionate to their knowledge of attending to such a complex process. Additional support should thus be made more readily available, for instance on directing attention towards workability instead of being preoccupied with obtaining information on cause of sick leave. Since the expectation of performing work tasks may serve to delegitimise sick leave, emphasising presence and workability should also be done with caution. The supervisors’ descriptions of the reciprocal nature of sickness absence follow-up also revealed that this practice is enmeshed with moral considerations and perhaps is as dependent on (inter)personal aspects as it is on formal guidelines in some cases. Although an individual approach to return-to-work is important, personal considerations may affect how employees are accommodated and result in unequal treatment in the return-to-work process. As such, this study provides insight into how supervisors´ knowledge of and response to return-to-work influence the process, underlining the importance of providing them support on how to approach employees with various diagnoses as their legislative responsibility increase.
